# Unique Aggregation of Sterigmatocystin in Water Yields Strong and Specific Circular Dichroism Response Allowing Highly Sensitive and Selective Monitoring of Bio-Relevant Interactions

**DOI:** 10.3390/md17110629

**Published:** 2019-11-06

**Authors:** Daniela Jakšić, Maja Šegvić Klarić, Ivo Crnolatac, Nataša Šijaković Vujičić, Vilko Smrečki, Marcin Górecki, Gennaro Pescitelli, Ivo Piantanida

**Affiliations:** 1 Faculty of Pharmacy and Biochemistry, Department of Microbiology, University of Zagreb, Schrottova 39, 10000 Zagreb, Croatia; djaksic@pharma.hr (D.J.); msegvic@pharma.hr (M.Š.K.); 2 Ruđer Bošković Institute, Bijenička c. 54, 10000 Zagreb, Croatia; Ivo.Crnolatac@irb.hr (I.C.); nsijakov@irb.hr (N.Š.V.);; 3 Department of Chemistry, University of Pisa, via Moruzzi 13, 56124 Pisa, Italy; gorecki_marcin@interia.pl (M.G.); gennaro.pescitelli@unipi.it (G.P.); 4 Institute of Organic Chemistry, Polish Academy of Sciences, Kasprzaka 44/52 St., 01-224 Warsaw, Poland

**Keywords:** toxins, *Aspergilli*, sterigmatocystin monitoring in water, chiral aggregation, non-covalent DNA binding

## Abstract

We demonstrated the hitherto unknown property of the mycotoxin sterigmatocystin (STC) to provide homogeneous solutions in aqueous medium by forming a unique aggregate type (not formed by analogous aflatoxins), characterized by exceptionally strong circular dichroism (CD) bands in the 300–400 nm range. Results showed that these CD bands do not originate from intrinsic STC chirality but are a specific property of a peculiar aggregation process similar to psi-DNA CD response. Transmission electron microscopy (TEM) experiments revealed a fine fiber network resembling a supramolecular gel structure with helical fibers. Thermodynamic studies of aggregates by differential scanning calorimetry (DSC) revealed high reversibility of the dominant aggregation process. We demonstrated that the novel STC psi-CD band at 345 nm could be applied at biorelevant conditions (100 nanomolar concentration) and even in marine-salt content conditions for specific and quantitative monitoring of STC. Also, we showed that STC strongly non-covalently interacts with ds-DNA with likely toxic effects, thus contrary to the previous belief requiring prior enzyme epoxidation.

## 1. Introduction

Large, non-charged aromatic molecules are commonly considered insoluble in water. However, it is well-known that many of these systems are strongly hydrophobic and tend to form aggregates and crystals based on π-stacking and dispersive interactions. In some cases, if a concentrated stock solution of a large aromatic molecule is prepared in an organic solvent miscible with water, and then a small aliquot is added to large bulk water, instant precipitation does not happen. Instead, a more or less stable homogeneous equilibrium state is formed, like in colloids or micelle-like constructs.

**Scheme 1:**
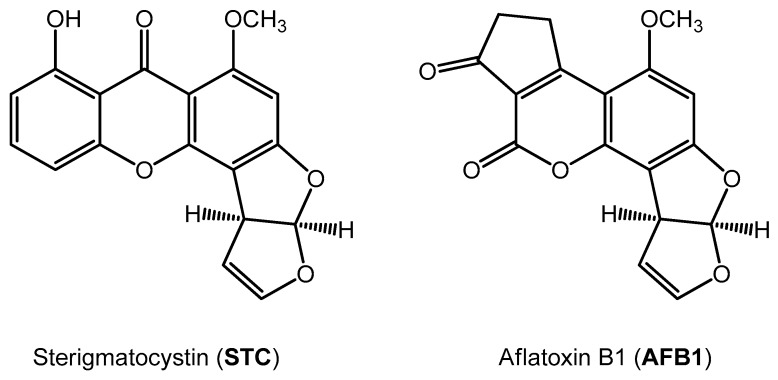
Studied mycotoxin sterigmatocystin (STC) and its close analogue aflatoxin B1 (AFB1).

In biological systems some naturally occurring large aromatic molecules play an important role, for instance, the family of aflatoxin-related mycotoxins [1] consists of several condensed aromatic systems (Scheme 1). Although being non-charged, most of them possess moieties (e.g., hydroxyl) with latent water solubility effect. Sterigmatocystin (STC, Scheme 1) is a secondary toxic metabolite of various filamentous fungi including the genera of *Aschersonia*, *Bipolaris*, *Botryotrichum*, *Chaetomium* and others, but most notably *Aspergilli* from the section *Versicolores* [2,3,4,5]. Mostly due to poor storage conditions, STC may be found in raw and processed food and feed [6,7,8,9,10,11,12], as well as in samples collected in indoor environments [3,11,13,14]. Some species from the section *Versicolores* are halo-tolerant and adapted to low-water activity so these properties may support their occurrence in the marine environment. Among these, the most often reported is the STC-producing species *A. sydowii,* associated with aspergillosis in coral reefs in the Caribbean, Pacific coasts of Colombia and the Ecuadorian Pacific coral reefs [15,16]. It was identified in fungal bloom which resulted from a great dust storm in Australia in 2009, rising the questions on its impact on the marine ecosystem [17]. In the Mediterranean region it was isolated from marine-bottom sediments, water, and calcareous shells of bivalve mollusks [18]. More recently, other species from the section *Versicolores*, i.e., *A. tabacinus* and *A. venenatus,* were also reported in marine environments [19]. The occurrence of primarily terrestrial fungus in marine environments encouraged the studies related to various bioactive metabolites derived from these organisms [20,21,22,23]. Despite its diffusion and biological relevance, to date there are relatively little data on the chemical characterization of STC. Structural similarity with aflatoxin B_1_ (AFB_1_), a well-known human (IA) carcinogen [1], prompted STC-related investigations in the context of hepatocarcinogenesis, and, according to the available data, it is classified as a type 2B carcinogen [24], and also listed as risk factor of gastric cancer and other disorders in the gastrointestinal system [25,26,27]. A high prevalence of STC in occupational and living indoor environments may contribute to various health hazards for the respiratory system [28]. A limited number of studies demonstrated the toxicity of STC in fish. Pathological alterations included hemorrhages and edema of the gills and hemorrhages and eosinophilic infiltrations in the hepatopancreas [29]. On Nile tilapia fish, the clastogenic effect of STC was indicated by the significant decrease of body weight and the increase in frequencies of micronucleated red blood cells and chromosomal aberrations in the kidney [30]. The complex toxicity-related metabolism consists of many transformations and interactions with various targets, including DNA non-covalent and covalent binding [31,32,33,34,35,36,37], enzyme-controlled epoxidation [38,39], and some others [40,41,42,43,44,45], of which many take place at water interface. These properties along with currently used analytical methods for AFB_1_ and STC determination (which include complicated procedures and application of organic solvent extractions [29,46]) prompted us to investigate the aqueous solubility properties of both mycotoxins (Scheme 1) and their water/lipophilic solvent distribution. The main aim was the development of a novel method for testing the presence of mentioned mycotoxins in aqueous solutions and with minimum sample processing. Unlike other aflatoxins, the literature data on chemical and physical properties of STC are insufficient and exclusively conducted using organic solvents (e.g., chloroform, methanol, acetonitrile) as the compound has been considered almost insoluble in water (at pH 7.5 present in nmol quantities) [47]. However, in this work by using a broad set of methods (UV and CD spectrophotometry, TEM, DSC, NMR, and DLS methods) we determined that STC is significantly more soluble and stable in aqueous solutions at biologically relevant concentrations ranging from 0.1 to 10 µM, in the presence of organic solvents as low as 0.1%. Most importantly, for the first time, we report STC-specific, exceptionally strong circular dichroism (CD) signal in the aqueous solution. The significance and possibilities for practical applications of these observations are presented and discussed in this paper.

## 2. Results and Discussion

### 2.1. Preparation and CD Spectra of Aqueous Solutions of STC

**Figure 1:**
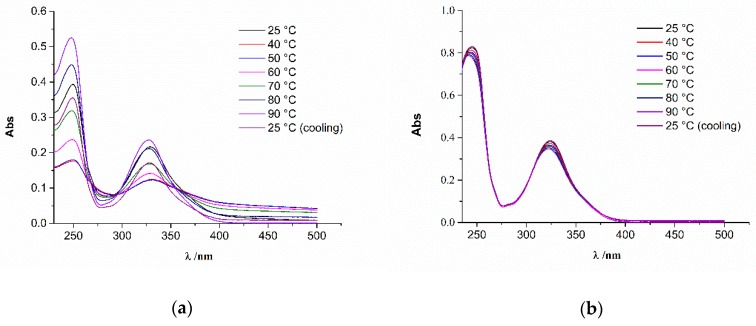
Temperature dependence of STC (c = 2.5 × 10^−5^ M) UV/Vis spectrum: (**a**) in aqueous solution (<1% acetonitrile); note the baseline drift (>400 nm) at low temperature strongly decreased upon heating; (**b**) in acetonitrile: water = 3:1; note only minor changes and stable baseline (>400 nm).

Because of the low water solubility of aflatoxin B_1_ and its metabolic precursor sterigmatocystin (STC), we prepared stock solutions in acetonitrile (in which both samples are well-soluble) at c = 5 × 10^–3^ M. The stock solutions were stored at −20 °C, and working aliquots were used daily for experiments. No precipitation, nor chemical degradation in the stock solutions were noticed. For the purpose of spectrophotometric characterization of AFB_1_ and STC chromophores in acetonitrile, UV/Vis (see Figure 1 and Figure S1 in Supplementary Materials) experiments were performed, agreeing well with literature data [5]. Further on, to test the limits of preparation of stable STC aqueous solutions, aliquots of acetonitrile stock solutions were systematically added to water, simultaneously monitoring the UV/Vis spectral properties. It was possible to prepare homogeneous aqueous solutions of STC stable at micromolar concentration and even at 10 µM concentration for a limited time. However, solutions of c(STC) > 5 µM shortly after preparation showed broadened absorption maxima and clear baseline drift in the UV/Vis spectrum (Figure 1, at 25 °C; >400 nm, at which STC does not absorb light), both changes being characteristic for large aggregate formation. It should be stressed that no opalescence or any kind of precipitate was observed, with the solution remaining completely transparent over several days. The incremental heating of STC aqueous solutions revealed (Figure 1a) a proportional increase of UV maxima and diminution of baseline drift (>400 nm) to zero, which could be attributed to the dissolving of STC aggregates into homogeneous STC solution. A parallel experiment with STC solution in a dominantly organic solvent (Figure 1b) showed very low sensitivity of STC UV/Vis spectrum to temperature, pointing out that in these conditions no aggregation occurs. Further, the cooling of aqueous STC solution back to 25 °C did not result in an immediate change of the UV/Vis spectrum, pointing out that aggregate formation is not an instantaneous process. The obtained results demonstrated the possibility of preparing stable STC aqueous solutions in biorelevant (micromolar) conditions, at variance to previously claimed STC insolubility in water.

**Figure 2:**
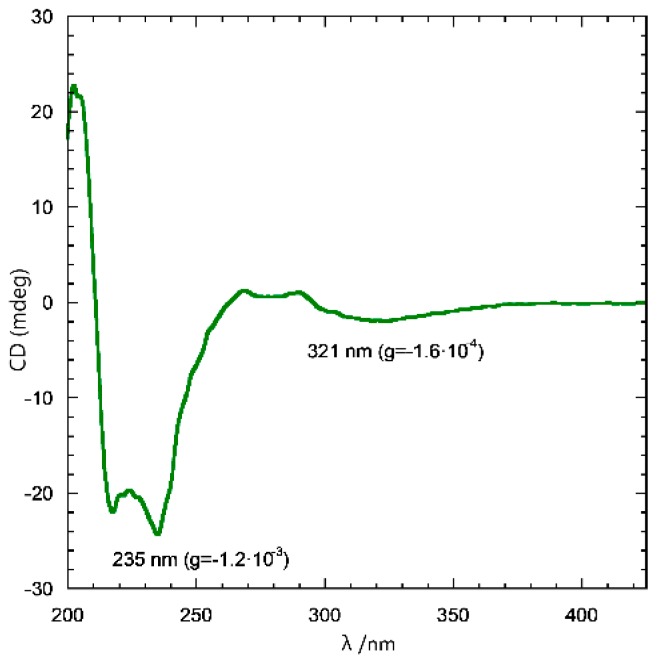
**The** CD spectrum of STC measured in acetonitrile solution (3 × 10^–4^ M, room temperature). To keep total abs < 2, in the range λ = 200–256 nm 0.1 cm pathlength, and λ = 256–400 nm 0.2 cm pathlength cuvette were used.

STC is intrinsically chiral, thus observed aggregation can in principle yield structurally well-defined chiral aggregates. For that purpose, we applied CD spectroscopy as a highly sensitive method for the study of chiral supramolecular systems [48,49]. The CD spectrum of STC in the acetonitrile solution showed two weak bands in correspondence with the main absorption bands at 240 and 320 nm (Figure 2). The measured *g*-factor (Δε/ε) for the 320 nm band was 10^–4^. The spectra did not display any sizable temperature dependence and varied linearly with concentration (Figure S2 in Supplementary Materials).

**Figure 3:**
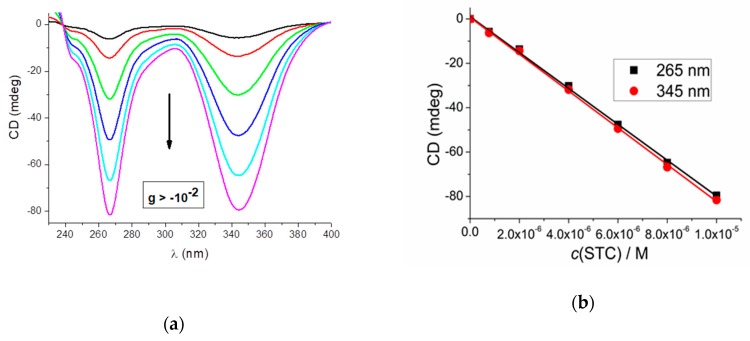
Concentration dependence of the STC CD spectrum in water in the range 10^–6^ to 10^–5^ M. Note exceptionally strong g-factors |Δε/ε| > 10^−2^.

Most intriguingly, the CD spectra of STC in aqueous solutions were of exceptionally strong intensity. They displayed two negative bands centered at 265 and 345 nm, both with *g*-factors above 10^–2^. The CD intensity varied proportionally with the STC concentration (Figure 3), meaning the Beer–Lambert law applied to CD was respected over the measured range of concentrations.

**Figure 4:**
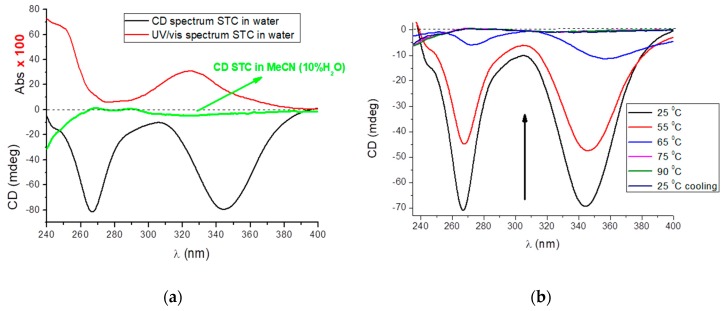
(**a**) Overlap of UV (red line, multiplied by 100) and CD spectrum of STC (black line), in water *c* (STC) = 1 × 10^−5^ M. Note that UV maximum (325 nm) does not match CD maxima (265 and 345 nm). For the CD spectrum of STC in acetonitrile (light green line, 10% H_2_O, multiplied by 2), note that the CD maximum at 345 perfectly matched the UV maximum. (**b**) Temperature dependence of CD spectrum of STC aqueous solution.

The difference in the CD profiles between the spectra measured in water and acetonitrile was first addressed to the aggregation-increased chiroptical response of STC molecules in water. However, we noticed that the CD maxima of aggregates (267 and 345 nm) did not correspond to the UV maxima (240 and 320 nm) (Figure 4a). Moreover, incremental heating of the STC solution completely cancelled the aggregate CD spectrum (Figure 4b), and, interestingly enough, the CD spectrum obtained in water at a high temperature matched well with the CD spectrum in acetonitrile (Figure S3, Supplementary Materials) attributed to non-aggregated STC species. Quite surprisingly, upon cooling back to 25 °C the CD bands at 265 and 345 nm were not recovered. These two observations pointed against the attribution of the CD spectrum observed in water as a standard aggregate CD, i.e., the typical CD signal obtained upon self-assembly of chiral monomeric units [48].

Rather, the behavior of STC in water suggested the presence of STC aggregates with unusual chiroptical properties. The linear concentration dependence of the CD spectrum (Figure 3) pointed out that the aggregate type does not change significantly within the 0.1–10 µM range and that a single, dominant aggregate form is observed. The intensity, shape, and appearance of the CD spectrum of STC in water is very reminiscent of the phenomenon known as psi-type (ψ-type) CD, which was observed a long time ago by Tinoco and Bustamante on toroidal DNA aggregates [50], and was later recognized in several other chiral large aggregates of various nature [51]. The distinctive characteristics of psi-type CD are the following [52,53]: (a) very large CD magnitudes, with 10–100-fold increase upon aggregation; (b) distorted band shapes, having no resemblance to the CD spectrum of the constituent molecules, non-conservative appearance (i.e., the total area under all positive and negative bands does not sum to zero), and no correspondence with absorption bands. The above peculiarities fit exactly our experimental observations about the behavior of STC in water. 

It has been theoretically formulated and experimentally demonstrated that psi-CD is related to particles endowed with a supramolecular chiral order, which have dimensions similar to the incident wavelength, that is, above 100 nm [52,53]. We show below that such large particles are in fact observed for STC. The main mechanism responsible for the extraordinary (chiro)optical properties of psi-type aggregates has been described as a collective excitation of the particle, mediated by long-range induced dipole couplings. This phenomenon has not yet been investigated by modern computational means, for which the nm-scale is still untreatable. However, taking advantage of the X-ray structure of STC [54], which we successfully reproduced from prepared single crystals (Figure S4, Supplementary Materials), we ran solid-state CD measurements and time-dependent density functional theory (TDDFT) calculations to verify whether the crystal packing might be associated with CD signals similar to those observed in water. Solid-state CD spectra measured on KCl pellets were relatively weak (Figure S5 in Supplementary Materials), with maximum *g*-factors around 10^–4^ and a shape reminiscent of that observed in acetonitrile solutions. On the contrary, there was no resemblance with the spectra measured in water, either in shape or intensity, demonstrating that the supramolecular architecture of water aggregates is not comparable to the crystal packing. To further confirm this conclusion, we calculated the CD spectrum associated with the crystal packing by employing a procedure developed before by some of us [55,56] (details are reported in the Supplementary Materials). The CD spectrum calculated for a small lattice portion (Figure S6, Supplementary Materials), comprising 15 STC units, is similar to that calculated for an isolated STC unit in the X-ray conformation. Thus, the crystal packing cannot be responsible for the strong and peculiar psi-type CD detected for water aggregates.

It must be stressed that neither aflatoxin AFB_1_ (Scheme 1), a very close analogue of STC, nor any other mycotoxin, did show any of the aforementioned aggregation properties yielding strong CD spectrum of similar strength or shape as STC. This could tentatively be attributed to the larger flat aromatic surface of STC with respect to AFB_1_, causing stronger aromatic stacking interactions; as well as to the additional –OH group at the terminal benzene ring, not only making STC more amphiphilic but also eventually taking part in the chiral organization of STC aggregate.

### 2.2. TEM Investigation

Transmission electron microscopy (TEM) is a powerful tool for imaging self-assembling molecules on the nanometer scale. It is the technique of choice for determining the structural building blocks of complex fluid systems, such as tubular, fibers, ribbons, vesicles, spherical, and helical structures [57,58]. Hierarchical self-assembly is a fundamental principle in nature, which gives rise to astonishing supramolecular architectures. 

**Figure 5:**
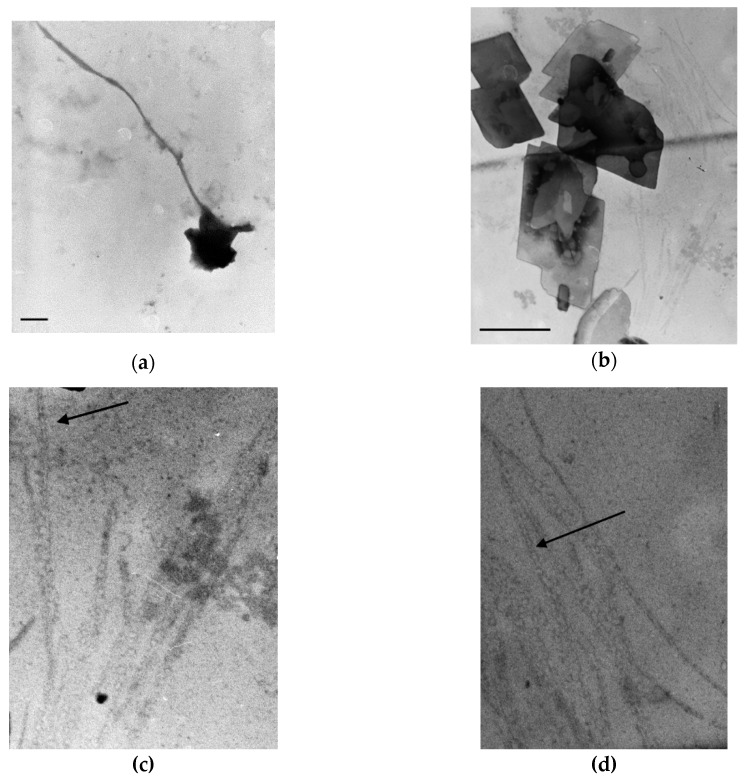
TEM images of the STC *c* = 2.5 × 10^−5^ M in water (stained with dipotassium polytungstate (PWK); bar = 500 nm). (**a**) A twisted tape of 80 nm diameter; (**b**) platelet-like crystals (diameters *d* around 200‒500 nm) and very tiny straight and helical fibers, d values of 6–12 nm; (**c**) and (**d**) helical fibers, enlarged part of image (**b**).

**Figure 6:**
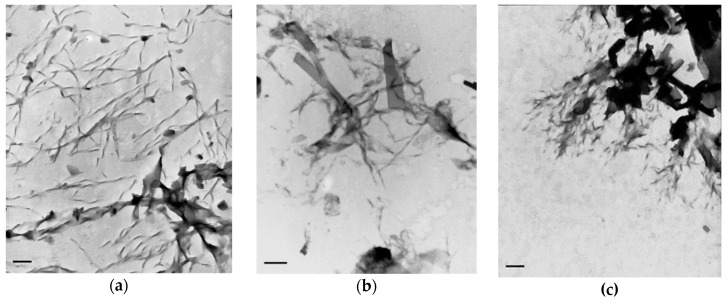
TEM images of the STC acetonitrile/water mixture (H_2_O:CH_3_CN = 1:1, *c* = 3 × 10^−3^ M, Pd shadowing, bar = 500 nm) showing different morphologies: (**a**) dominantly twisted ribbons mostly of left-handed helicity (d values of 60–100 nm), (**b**) mostly straight ribbons (d values of 200–500 nm) and (**c**) platelet-like crystals (d values of 200–1000 nm) combined with ribbons.

The TEM micrographs of STC samples in pure water and acetonitrile/water mixtures showed distinct chiral and achiral morphologies such as fibers, helical fibers, twisted tapes, nanotubules, straight fibers, straight tapes, and larger platelet-like crystals, often coexisting in the same sample. The TEM image of the STC water solution (c = 2.5 × 10^−5^ M, Figure 5 and Figure S7, Supplementary Materials) shows the simultaneous presence of larger platelet-like crystals (diameters (d) around 200–500 nm), twisted tapes (d values of 80 nm) and small nanofibers (d values of 6–12 nm; Figure 5b,c). The TEM image of the STC turbid viscous solution in acetonitrile/water mixture (ratio 1:1; c = 3 × 10^−3^ M) reveals the presence of three distinct morphologies, straight ribbons (d values of 200–500 nm), twisted ribbons mostly of left-handed helicity (d values of 60–100 nm), and platelet-like crystals (d values of 200–1000 nm; Figure 6). The sample with higher water content in acetonitrile/water mixture (ratio 1:2) showed the formation of larger tubules with d values in the range of 60–500 nm (Figure S8 in Supplementary Materials). The TEM observations show that STC molecules self-organize in different supramolecular aggregates including some with chiral morphology (twisted ribbons, helical fibers) under the solvent conditions used.

When tracking STC concentration– and solvent–dependent morphologies, we observed elongated fibers and tapes at lower concentrations of STC, which further developed into twisted ribbons and nanotube superstructures at increased concentrations. Nevertheless, the self-assembled STC nanostructured supramolecular aggregates are always present in equilibrium with platelet-like crystals whose formation is observed as the dominant process. 

We also prepared single crystals by slow evaporation of STC acetonitrile solution and X-ray single-crystal diffraction analysis revealed the same crystal packing as previously reported [54]. In the crystal, STC molecules are closely stacked by ideal orientation and distances for intensive aromatic π-π interactions (Figure S4, Supplementary Materials), thus agreeing well with the proposed major driving force of aggregation in aqueous solutions. 

However, in water, the crystallization/precipitation is not the only outcome of STC aggregation, since at micromolar and lower concentrations STC forms a large variety of non-crystalline entities such as fibers, which are part of homogeneous solution exhibiting the exceptional CD response discussed above. For a better characterization of aggregate size distribution, we also performed dynamic light scattering (DLS) analysis in 10 µM aqueous solutions (Figure S9, Supplementary Materials). The dominant particle size population for all measurements was within 100–600 nm, whereby the presence of a minor population (about 1000 nm size) was proportional to STC concentration increase. The heating of a sample to 60 °C yielded a very sharp peak of size population within 400–600 nm, which can be attributed to the dominant aggregate type. At higher temperatures no signal was measured, agreeing well with the complete dissolving of STC aggregates to single molecules. Thus, DLS measurements confirm the existence of particles with size > 100 nm which can be responsible for psi-type CD as discussed in the CD results section (Section 2.1). 

### 2.3. Thermodynamic Profiling of STC Aggregates by Differential Scanning Calorimetry (DSC)

Differential scanning calorimetry (DSC) measures the excess heat capacity of a solution (C_p_) as a function of temperature, and the melting of the non-covalent construct is usually recognized as a more or less sharp endothermic peak centered at T_m_ and the maximum in C_p_ occurs directly at T_m_. Thus, DSC was the ideal method to directly study the thermodynamics of STC aggregation. 

**Figure 7:**
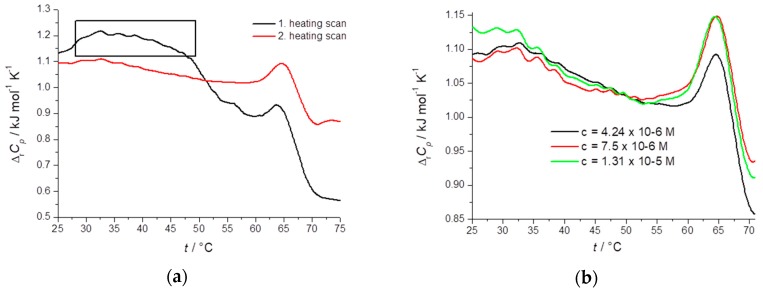
Change of molar heat capacity as a function of temperature scan of STC from 10 °C to 100 °C at heating rate 1 °C min^−1^. (**a**) First and second scan at a concentration of 4.24 × 10^–6^ M. Note the non-reversible transition in *T*_m_ = 25–55 °C range marked by square. (**b**) Second scan at three different STC concentrations (see spectrum label) within the range of CD experiment (Figure 3 and Figure 4).

In agreement with the TEM experiments described above (Figure 5 and Figure 6), DSC results demonstrated the simultaneous presence of different polymorphic aggregates (e.g., nanofibers and tapes) in aqueous STC solutions. DSC experiments performed under the same conditions as for CD study (water solutions, 4–13 µM range, Figure 7) revealed a well-defined melting temperature T_m_ = 66 °C, which did not depend on STC concentration. However, in the first heating cycle the DSC heating curves for STC sample showed two endothermic transitions (Figure 7a), whereby a broad endothermic transition (*T*_m_ = 25–55 °C) was not reversible in any of subsequent heating cycles, while the second endothermic transition at *T*_m_ = 66 °C was excellently reversible even in several consecutive heating and cooling cycles (Figure 7b). Hence, the DSC-observed transition for the STC water solution at 66 °C originated from the melting of highly reversible aggregates. However, the lower-temperature transition (25–55 °C) may originate from the melting of the less stable and kinetically slow-forming aggregates, which could be correlated to the reduction of the signal intensity in the CD spectrum in the range of 25 to 65 °C (Figure 4). Moreover, both, DSC lower-temperature transition (25–55 °C) and CD bands at 265 and 345 nm were not recovered upon cooling (even after a week at +8 °C), pointing out that peculiar aggregate-chirality is either a very slow or kinetically trapped process. The small differences in enthalpy change of the dominant transition (Table 1) observed in consecutive DSC cycles are likely originated from different ratios of low-stability aggregates present in the sample.

### 2.4. NMR Study of STC Aggregation

**Figure 8:**
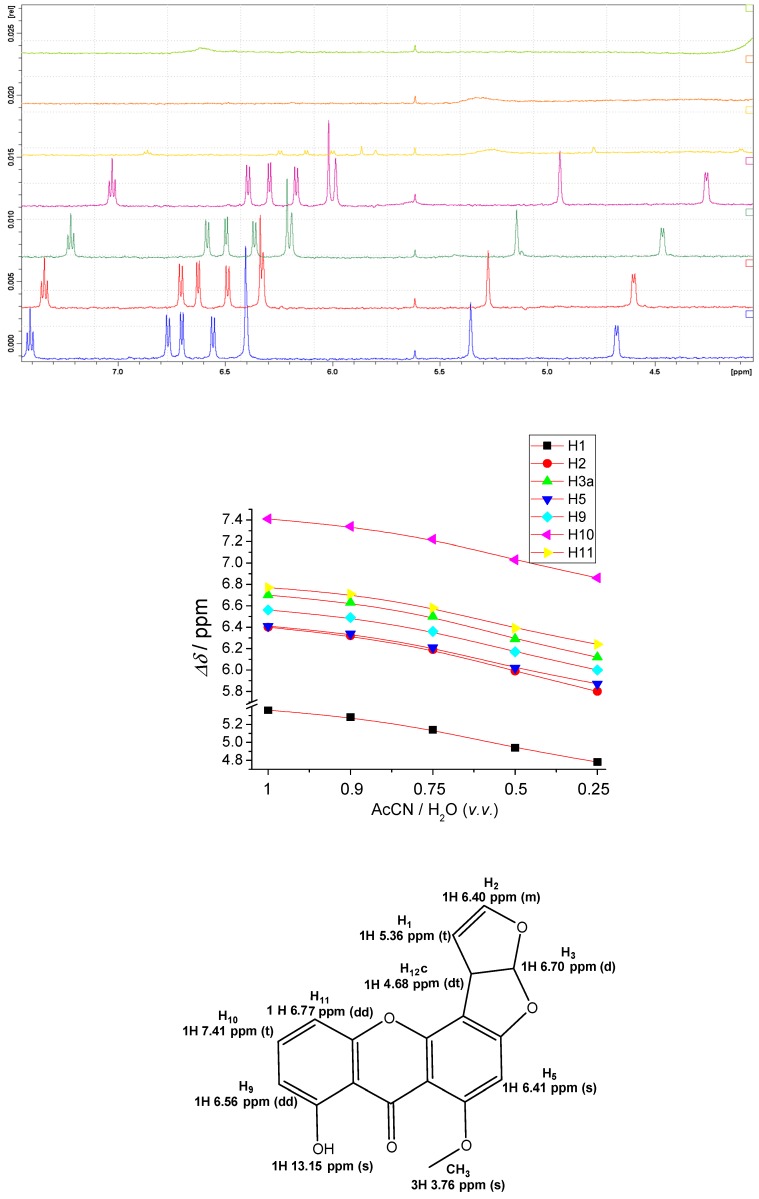
^1^H NMR spectra of STC in various acetonitrile/water mixtures (*v.v.* CD_3_CN/D_2_O ratios from bottom to top: 100%; 75%; 50%; 25%; 10%; 5% CD_3_CN) *c*(STC) = 2.5 × 10^−4^ M; with TMS as an external standard (sealed capillary). The complete ^1^H and ^13^C assignment is presented in Table S1 in Supplementary Materials.

The formation of STC aggregates was expected to be driven by stacking interactions between aromatic rings. In such well-oriented systems usually ^1^H NMR study could be very informative, revealing π-π interaction-induced changes of proton signals, such as upfield *Δδ* shifts and signal broadening. According to previous data, the NMR spectrum of STC in *d*^3^-acetonitrile (Figure 8, bottom spectrum) was attributed to isolated STC molecules devoid of any intramolecular interaction.

The increasing ratio of water with respect to acetonitrile resulted in the upfield shift of all proton signals (Figure 8), whereby finally, at acetonitrile: water ratios < 25% no proton signals of STC were detectable, although the solution was still transparent and no precipitation was observed. The observed changes in ^1^H NMR spectra agree with previous UV/Vis and CD experiments (Figure 1 and Figure 3, Figure S1 in Supplementary Materials), whereby upfield shifting and minor broadening of proton signals is typical for aromatic stacking interactions (again in agreement with X-ray structure of crystal [54]).

The stepwise heating of the sample CH_3_CN/H_2_O = 0.05 (Figure S10, Supplementary Materials) resulted in partial recovery of ^1^H NMR signals only at 60 °C and above, supporting additionally the non-covalent interactions in aggregates, as well as the existence of a crucial temperature for the major aggregate disintegration (see DSC, DLS, and CD results).

### 2.5. Study of STC Aggregation in Biorelevant Conditions

Motivated by uniquely strong CD response to STC aggregation, as well as the applicability of these results in sensitive and specific mycotoxin detection, we studied in detail the STC aggregation in biorelevant conditions.

**Figure 9:**
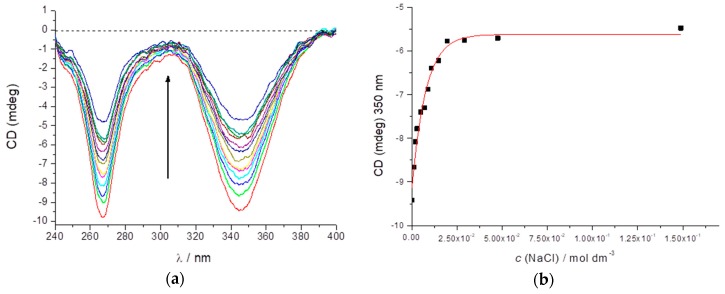
(**a**) Decrease of the STC CD spectrum intensity (*c*(STC)=1 × 10^−6^ M) with increasing NaCl concentration. (**b**) Dependence of a 350 nm band intensity at 350 nm for the *c*(NaCl) = 10^−5^ to 1.5 × 10^−1^ M.

The STC-aggregated CD spectrum in buffered conditions (pH 7, Na cacodylate buffer, *I* = 0.05 M) had an identical shape to the CD spectrum in pure water but with slightly lower intensity. However, physiological and marine-salt conditions contain significantly higher NaCl concentrations. The systematic variation of *c*(NaCl) in solution (Figure 9) yielded a strong CD decrease, but the general shape of the psi-type CD spectrum was retained. Such an effect of NaCl is counterintuitive because it is expected in general to promote the aggregation of hydrophobic molecules in water; it seems however that the chiral psi-type of aggregate is not favored at the high salt concentrations with respect to other, less CD-active aggregates or even crystalline forms although it still survives to some extent. 

**Figure 10:**
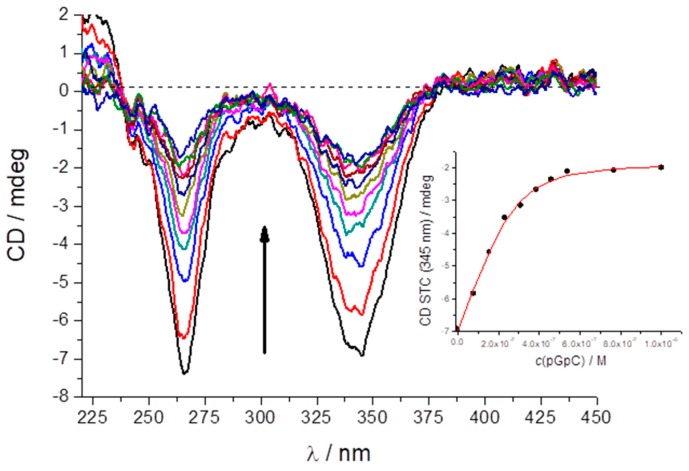
CD titration of STC (7.7 × 10^−7^ M) with GC-DNA at pH 7.0, sodium cacodylate buffer, *I* = 0.05 M, 25 °C. Inset: dependence of STC CD 345 nm maximum intensity upon titration with GC-DNA.

Further, the main biorelevant target on which STC and other related mycotoxins, e.g., aflatoxins, base their toxicity is cellular DNA. Although mostly enzyme-induced covalent binding to DNA-guanine was determined for both STC [37] and related aflatoxin AFB_1_ [33], determined structures clearly showed intercalation of aromatic mycotoxin between GC-basepairs of DNA, thus suggesting that non-covalent interactions (aromatic stacking) may precede covalent binding. Therefore, we decided to study the impact of GC-DNA addition to STC aggregate properties in an aqueous solution by monitoring the changes in the CD spectrum of aggregate (Figure 10). For that purpose, particularly convenient was the dominant CD band of STC aggregate at 345 nm since GC-DNA does not absorb light at wavelengths > 300 nm and thus cannot interfere (Figure S11 in Supplementary Materials). The addition of GC-DNA strongly decreased the intensity of the CD band of STC aggregate at 345 nm, whereby non-linear fitting of the experimental data (Figure 10, inset) excellently agreed with the Scatchard equation [59], commonly used for the analysis of small molecule-DNA binding affinity. The results could be attributed to STC aggregate dissociation caused by single STC molecules binding into DNA. Under the presumption of psi-type STC aggregate as a starting entity in the solution and a single STC molecule bound to DNA as the dominant DNA-binding mode, the binding constant *K_s_* ≈ 0.1 µM range could be estimated. 

Additional experiments demonstrated very similar changes in the STC CD spectrum upon the addition of AT-DNA (Figure S12 in Supplementary Materials). A detailed inspection of the CD spectrum of GC-DNA showed that the intensity of all DNA bands decreased upon STC addition (Figure S11 in Supplementary Materials), especially looking at CD maxima at 250 and 275 nm where the STC CD spectrum has little influence. Such changes point to a structural modification of DNA helix upon STC binding. Further, we performed thermal denaturation experiments (Figure S13 in Supplementary Materials), which showed strong stabilization of AT-DNA by STC addition (for ratios r[STC]/[DNA] = 0.5 and 1, Δ*T*_m_ = +13 °C and +20 °C, respectively). It was not possible to perform the denaturation experiment with GC-DNA because of the high *T*_m_ > 95 °C of free GC-DNA, which with STC addition would exceed water boiling point. Thus, the obtained results consistently support non-covalent interaction of STC with ds-DNAs; the strong thermal stabilization and the planar aromatic structure of STC suggest intercalation between basepairs as the most probable binding mode. Although for the exact determination of non-covalent interactions between STC and DNA further studies are needed, for the first time we showed that STC can non-covalently interact with ds-DNA, which opens the possibility that STC toxicity is not only caused by an enzyme-driven chemical reaction with guanine [33,37], but also can be caused by STC non-covalent interactions; for instance as found for some other toxins intercalating into DNA [60]. 

## 3. Materials and Methods 

### 3.1. Chemicals

STC standard, dimethyl sulfoxide (DMSO), methanol and acetonitrile (HPLC grade), as well as poly dGdC – poly dGdC and poly dAdT – poly dAdT and buffers were purchased from Sigma-Aldrich (Deisenhofen, Germany). Deionised water was produced by Millipore Milli-Q Gradient A10 water purification equipment (Merck Millipore, Croatia). All other chemicals were from Kemika (Zagreb, Croatia). The concentration of DNAs was determined spectroscopically by molar extinction coefficients provided by producer (*ε*_262_ = 6600 for AT-DNA; *ε*_254_ = 8400 for GC-DNA), whereby it is expressed as *c* (DNA phosphates).

### 3.2. UV-Vis Spectroscopy

UV-Vis spectrophotometer Varian Cary 100Bio (Varian Inc, Palo Alto, CA, USA) was used to measure absorption spectra of STC in methanol: water (3:1 *v*/*w*) acetonitrile: water (3:1 *v*/*w*), mQ-H_2_O and sodium cacodylate buffer (*I* = 0.05 M, pH = 7) at various temperature ranges. Stock solution of STC was prepared by dissolving STC in DMSO or acetonitrile.

### 3.3. CD Spectroscopy 

CD spectra were recorded on JASCO J-815 spectropolarimeter (Jasco Inc., Tokyo, Japan) at defined temperature using 0.1, 0.2 and 1 cm path quartz cuvettes with a scanning speed of 200 nm/min; each spectrum was the result of at least three accumulations. A solvent background was subtracted from each spectrum. The STC solutions for measurements were prepared by adding small aliquots of STC stock solution (in DMSO or acetonitrile) to an aqueous solution. An attempt to directly dissolve STC in water yielded colloid solutions not adequate for CD measurements. Solid-state CD spectra were measured using the KCl pellet technique [61]. About 0.1 mg of STC crystals were mixed and ground with 100 mg of KCl, then this mixture was used to prepare the pellet under high pressure (10 ton cm^–2^). The CD spectra obtained by rotating the pellet around the incident axis direction and/or by flipping around the vertical axis were all consistent, revealing that the contribution from linear dichroism artifacts was negligible. 

### 3.4. Differential Scanning Calorimetry (DSC)

Differential scanning calorimetry (DSC) was used to investigate the melting of STC using an instrument Nano DSC from TA Instruments (New Castle, DE, USA). The DSC scans of STC were made against a mQ-H_2_O from 10 °C to 100 °C at a heating rate of 1 or 2 °C min^−1^. 

The stock solution of STC was prepared by dissolving 0.52 mg STC in mQ-H_2_O by heating in an ultrasonic bath and checking the STC concentration by molar extinction coefficient (ε(324 nm) = 21313 mol^−1^dm^3^cm^−1^). The lowest STC concentration (4.24 × 10^−6^ M) was additionally filtered through 0.45 µm Millipore filter to see if this would have an impact on DSC measurements.

DSC measures the excess heat capacity of a solution (C_p_) as a function of temperature. The melting is recognized as a sharp endothermic peak centred at T_m_ and the maximum in C_p_ occurs directly at T_m_ (Figure 7 and Figure 8). Integration of the C_p_ versus T curve yields the calorimetric enthalpy (∆_r_H_c_) and the shift in the baseline yields the ∆_r_C_p_.

The calorimetric enthalpy is the total enthalpy change including the contributions from all processes and determined independently of any model while the corresponding ∆_r_H_vH_ assumes a simple two-state transition.

### 3.5. DLS Method

Measurements were performed on Zetasizer Nano ZS (Malvern Panalytical Ltd., Malvern, UK) at defined temperature by measuring the same samples used in CD measurements.

### 3.6. Transmission Electron Microscope (TEM)

A sample of the tested substance was placed in a test tube and the requisite solvent was added. After addition, the mixture was gently heated until the substance dissolved, then allowed to cool naturally to room temperature. The morphologies of self-assembled structures of STC formed in different solvent mixtures were observed by transmission electron microscope Zeiss EM 10A (Carl Zeiss AG, Oberkochen, Germany) operated at an accelerating voltage of 60 kV. A small amount of sample was placed on a carbon-coated grid (copper, 100 mesh). The sample was negatively stained with PWK (dipotassium polytungstate) or Pd shadowing. 

### 3.7. NMR Experiments

All NMR experiments were carried out by using Bruker Avance 600 (Bruker BioSpin Corporation, Bellerica, MA, USA) spectrometer (600,13 MHz, ^1^H). Samples in different mixtures of CD_3_CN and D_2_O solutions were recorded in 5 mm NMR tubes at 298 K (if not indicated differently). Chemical shifts in parts per million were referenced to TMS as internal standard – a capillary with TMS in acetone-d_6_ was put into the NMR tube. ^1^H spectra were recorded using a broadband-observed (BBO; BB, ^1^H; outer coil tuned for ^1^H) probe with a built-in z-gradient coil. Typically, spectra at a spectral width of 9 kHz and a digital resolution of 1.1 Hz per point were measured with 1024 scans. All experimental data were zero-filled to quadruple the number of experimental points. All spectra were baseline-corrected using automatic baseline correction. The software TopSpin (Bruker BioSpin Corporation, Bellerica, MA, USA) version 2.1 was used for all acquisition and processing.

## 4. Conclusions

We showed that, contrary to previous notions, STC can dissolve in water as a homogeneous solution at biologically relevant (micromolar) concentrations. The aqueous solubility of STC is based on a reversible aggregation process. Using various spectroscopic, calorimetric and microscopic methods we proved that STC molecules form a variety of different aggregate types. 

Most intriguingly, STC aggregates showed a unique and exceptionally strong CD response in the 250–400 nm range, not observed for any related mycotoxin analogue (aflatoxins and other). We propose that such large chiral objects are responsible for extraordinary chiroptical properties which can be classified as psi-type CD bands. 

Such exceptionally strong CD signal opened a new approach to specific STC detection and quantification, in line with European Food Safety Authority (EFSA) call (2013) [29] requiring broader investigation of the STC content in food and feed as the available data was scarce and left censored. Recent improvements in already used techniques (LC/MS technique; new immunoaffinity columns [8,9]; fluorescent sensor based on carbon dots-embedded molecularly imprinted polymer (CDs@MIP) [46]) still require laborious procedures like extraction with organic solvents, various clean-up procedures, addition of expensive internal standard (isotopically labelled STC), to compensate for matrix effects, etc. Here, we demonstrated that aqueous STC solutions (with no sample preparation or processing) could be directly studied at low (submicromolar) concentrations to monitor and, more importantly, quantify STC interactions with the main biological target (DNA). In addition, we demonstrated that STC strongly non-covalently binds to DNA (well-known highly toxic and carcinogenic event), for which, contrary to previous belief [34], enzyme epoxidation is not required. Thus, here presented data offers a novel tool for monitoring STC, not only as one of the under-investigated mycotoxins but also as the precursor of various aflatoxins, and numerous related mycotoxin metabolites.

Future prospects for applications of here presented results span over numerous other fields aside mycotoxin analytics. The new information about the self-assembly of STC molecules provided insight in processes of complementary intermolecular hydrophobic and aromatic stacking interactions and demonstrated an effective pathway to obtain chiral nanostructures derived by the non-covalent aggregation of the same small molecule.

## Figures and Tables

**Table 1:** Calorimetry DSC data for the compound STC obtained during heating from 10 to 100 °C with the rate of 1 °C min^−1^.

*c*(STC)/M	Scan	*T*_max_/°C	Δ_r_*H*_c_/kJ mol^−1^	Δ_r_*S*_c_/J K^−1^ mol^−1^
^1^ 4.24 × 10^−6^	#1	65.7	57.1	168.5
	#2	66.1	40.2	118.5
7.5 × 10^−6^	#1	66.3	27.2	80.0
	#2	65.9	21.8	64.3
	#3	65.2	31.1	91.9
	#4	64.8	32.7	96.7
1.31 × 10^−5^	#1	65.9	21.7	64.1
	#2	65.9	16.6	49.0

^1^ Pre-filltered solution, thus large aggregates were removed.

## Supplementary Materials

The following are available online at https://www.mdpi.com/1660-3397/17/11/629/s1, Figure S1: Additional UV spectra, Figures S2 and S3: Additional CD spectra in water and acetonitrile at different temperature, Figure S4: X-ray geometries, Figure S5: Solid-state CD spectra, Figure S6: CD calculations on lattice portions, Figures S7 and S8: TEM images, Figure S9: DLS measurements, Figure S10: NMR spectra in acetonitrile:water, Figure S11–13 Additional CD spectra of STC and ds-DNA interaction, Table S1: ^1^H NMR data.
