# Supplementary material for: Unique Aggregation of Sterigmatocystin in Water Yields Strong and Specific Circular Dichroism Response Allowing Highly Sensitive and Selective Monitoring of Bio-Relevant Interactions
Source: Mar Drugs. 2019 Nov 6;17(11):629. doi: 10.3390/md17110629 (PMC6891739; doi:10.3390/md17110629)
Supplement: Supplementary file 1 [file marinedrugs-17-00629-s001.zip › marinedrugs-625954-supplementary.pdf]

Article

Unique Aggregation of Sterigmatocystin in Water Yields Strong and Specific Circular Dichroism Response Allowing Highly Sensitive and Selective Monitoring of Bio-Relevant Interactions

Daniela Jakšić [1], Maja Šegvić Klarić [1], Ivo Crnolatac [2], Nataša Šijaković Vujičić [2], Vilko Smrečki [2], Marcin Górecki [3,4], Gennaro Pescitelli [3] and Ivo Piantanida [2,*]

[1] Faculty of Pharmacy and Biochemistry, Department of Microbiology, University of Zagreb, Schrottova 39, 10000 Zagreb, Croatia; djaksic@pharma.hr (D.J.); msegvic@pharma.hr (M.S.K.)

[2] Ruđer Bošković Institute, Bijenička c. 54, 10000 Zagreb, Croatia; Ivo.Crnolatac@irb.hr (I.C.); nsijakov@irb.hr (N.S.V.); Vilko.Smrecki@irb.hr (V.S.)

[3] Department of Chemistry, University of Pisa, via Moruzzi 13, 56124 Pisa, Italy; gorecki_marcin@interia.pl (M.G.); gennaro.pescitelli@unipi.it (G.P.)

[4] Institute of Organic Chemistry, Polish Academy of Sciences, Kasprzaka 44/52 St., 01-224, Warsaw, Poland

* Correspondence: Ivo.Piantanida@irb.hr; Tel.: +385 1 4571326

Received: 10 October 2019; Accepted: 4 November 2019; Published: date

Spectrophotometric experiments

(a)

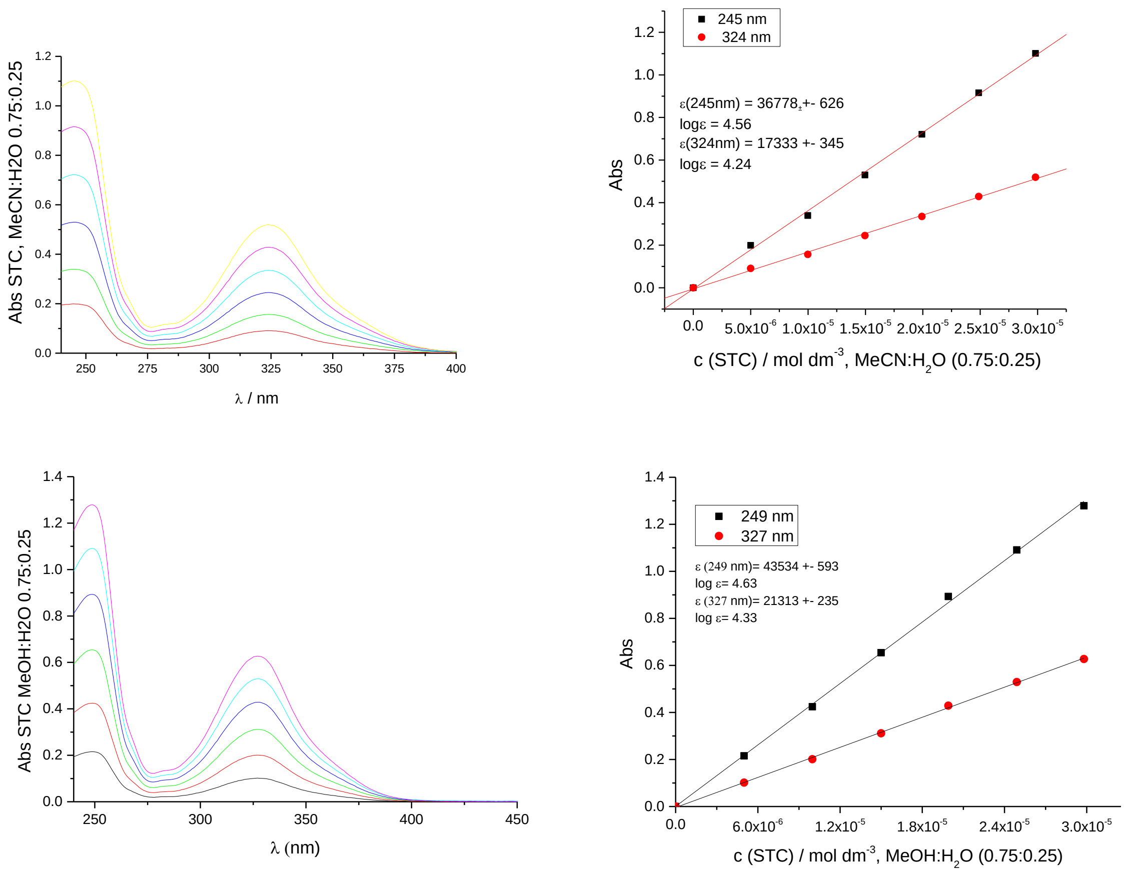

(b)

Figure S1. UV spectra of STC in concentrations ranging from 5×10^{-6} to 3×10^{-5} M in acetonitrile/water 0.75:0.25; (**a**) and methanol/water 0.75:0.25 (**b**) with corresponding calibration lines. Absorbance of STC is proportional to its concentration within the used concentration range.

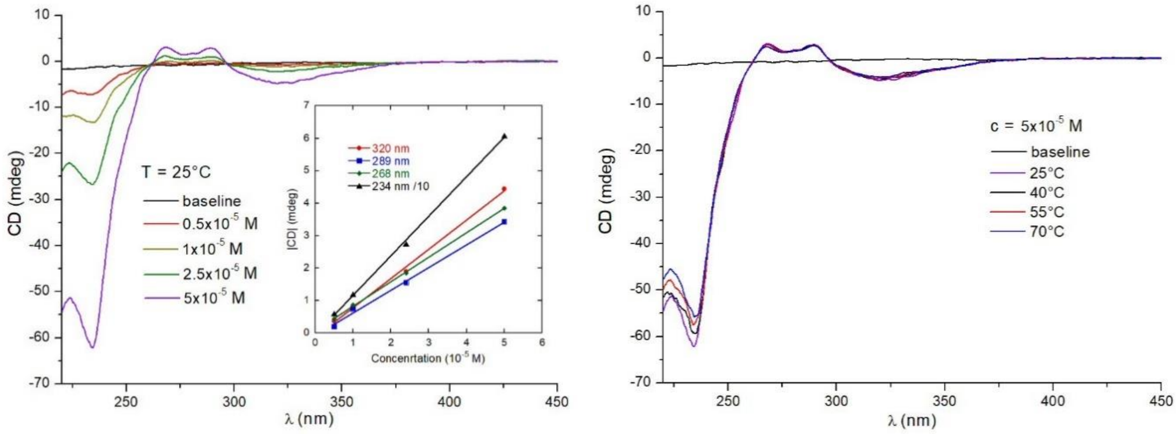

Figure S2. Concentration (left) and temperature dependence (right) of CD spectra of STC in acetonitrile. Cell pathlength 1 cm. Samples were prepared starting from a stock solution of STC 10^{-2} M in acetonitrile. The baseline spectrum was recorded on acetonitrile in the same cell. The inset in the left panel shows the linear dependence of the CD maxima on the concentration.

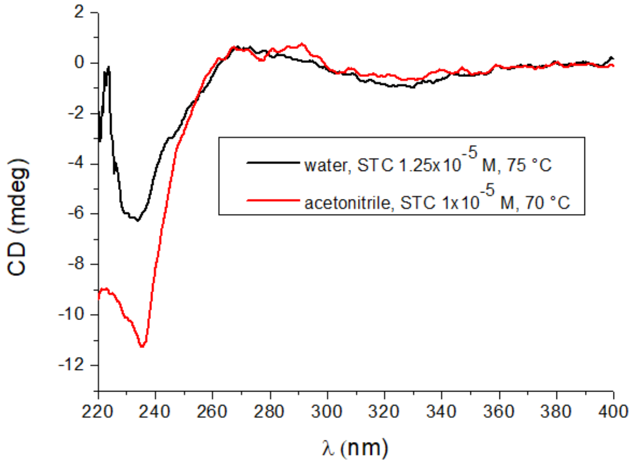

Figure S3. Comparison between the CD spectrum of STC in acetonitrile at 70°C and the CD spectrum of STC in water at 75 °C at the same concentrations.

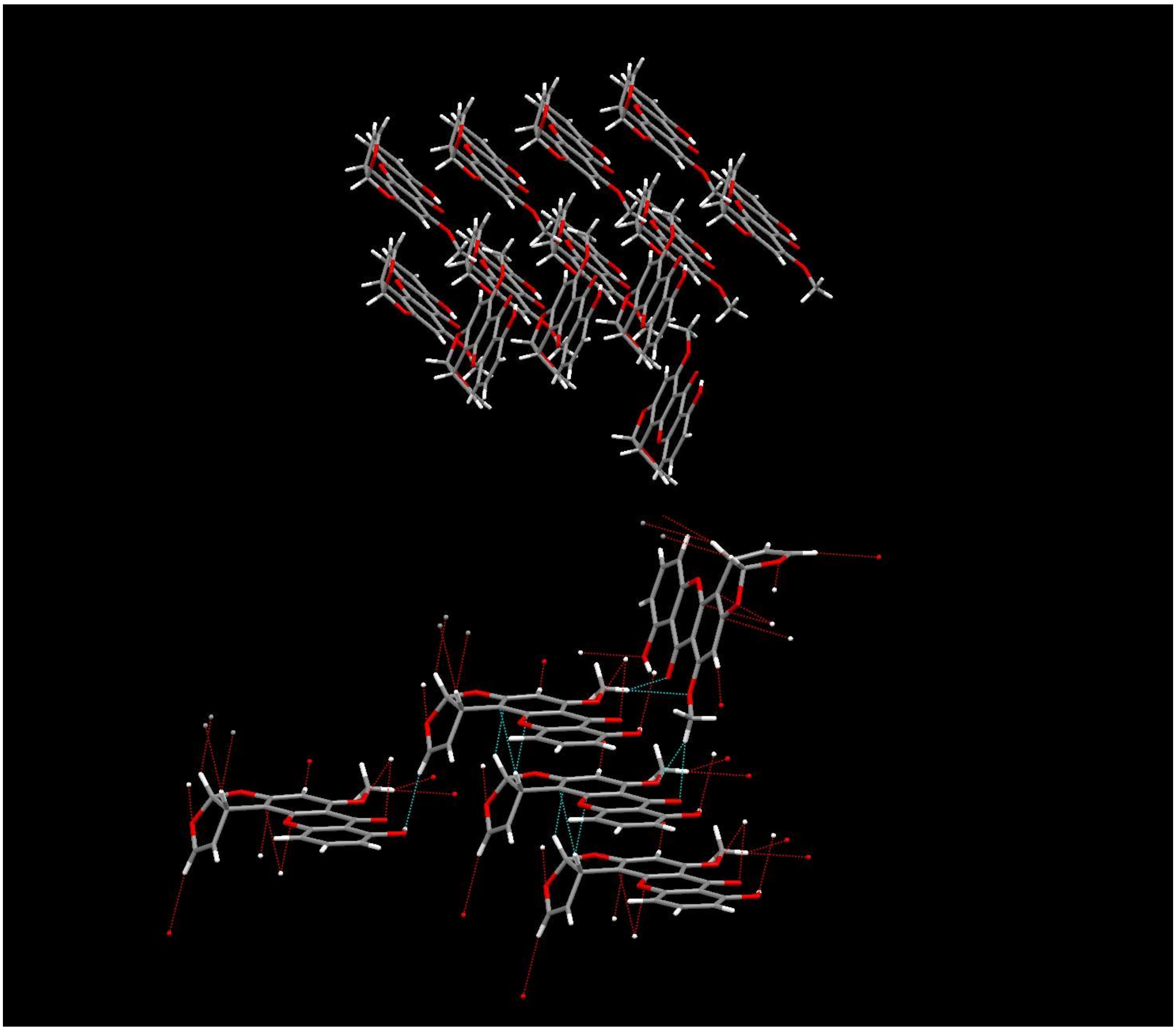

Figure S4. X-ray single crystal diffraction analysis of single crystals prepared by slow evaporation of STC acetonitrile/H_2O (1:1) solution revealed the same crystal packing as previously reported.[1]

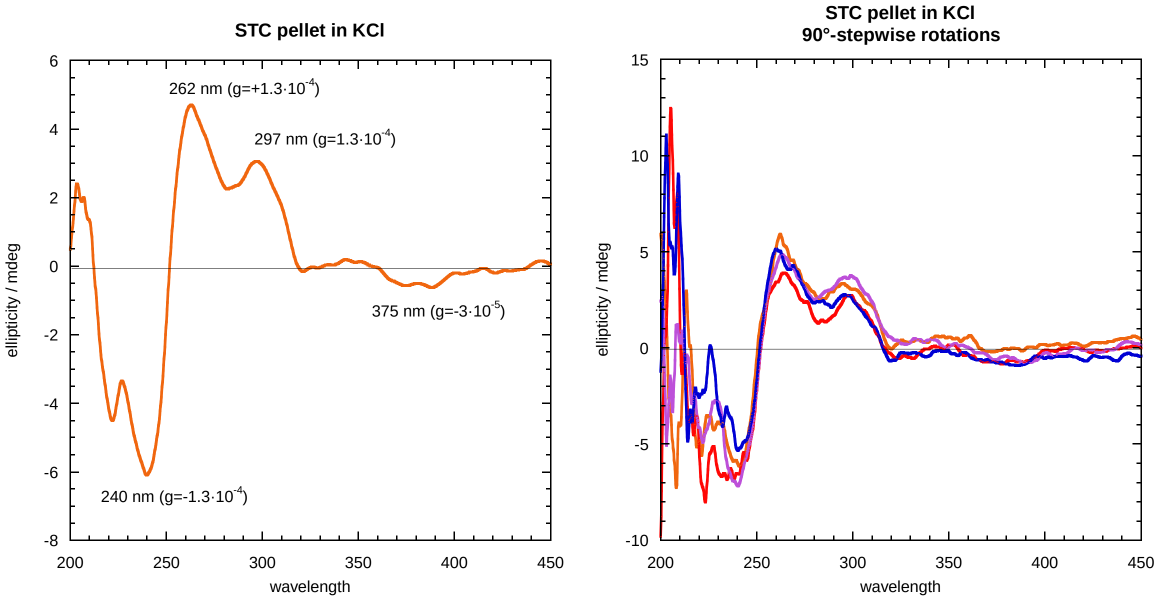

Figure S5. Solid-state CD spectra of STC dispersed in KCl matrix measured with the pellet technique (see main text, Experimental Section). Left: final spectrum obtained by averaging different orientations. Relevant *g*-values are reported for selected maxima and minima. Right: component spectra obtained upon rotating the pellet by 90° stepwise rotations around the incident light direction.

CD calculations on lattice portions of sterigmatocystin

To calculate the CD signal associated with STC in its crystalline state, we employed an approach based on the evaluation and summation of two-body (or pairwise) terms between "first-sphere" lattice neighbors. This approach was successfully applied to reproduce solid-state CD spectra in the presence of weak or moderate intermolecular exciton couplings.[2]

Examination of the crystal lattice of STC (Figure S3 and S4, left) reveals that each "probe" molecule (yellow in Figure S7) is surrounded by 14 effective closest neighbors with shortest distance between heavy atoms below 3.5Å. Some of the 20 possible "dimers" obtained either by translation or roto-translation are equivalent because of symmetry reasons. CD spectra were calculated for all possible "dimers" at the CAM-B3LYP/SVP level and averaged, then compared with the spectrum calculated at the same level for the "isolated" probe (Figure S4, right). The two spectra are very similar to each other, witnessing that the average perturbation provided by lattice neighbours is negligible. This result is due to the alignment observed among the molecules in the lattice, either in a head-to-head or in a head-to-tail arrangement, which leads to small intermolecular CD exciton coupling effects. More importantly, it demonstrates that the strong CD signal produced by aggregates in water is associated with a supramolecular architecture well distinct from the crystal lattice.

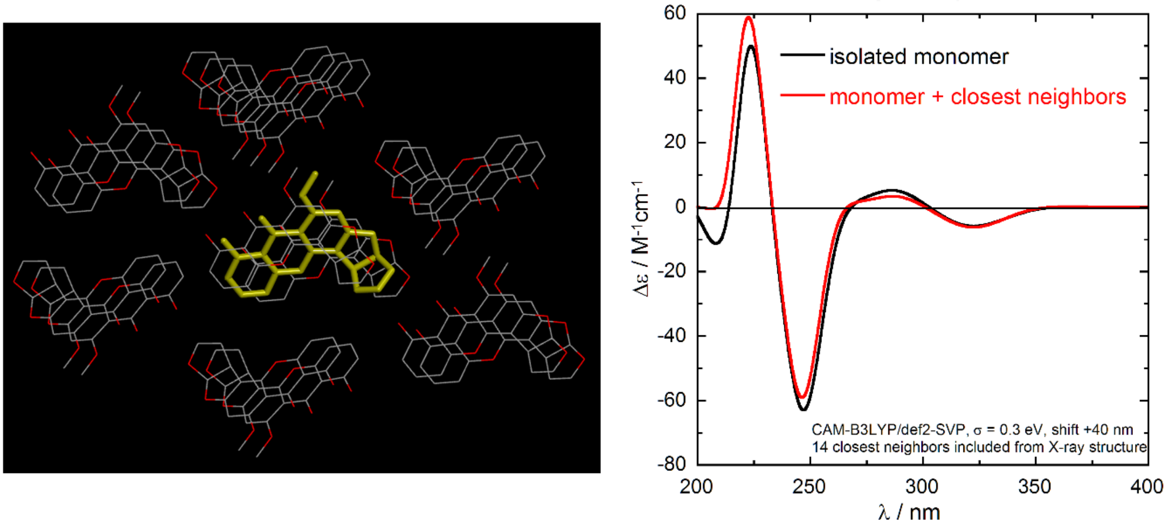

Figure S6. Left: a probe molecule of STC (in yellow) surrounded by its closest neighbors in the crystal lattice. Right: comparison between the CD spectrum calculated for the isolated probe molecule of STC and the average of 14 spectra calculated for the dimers between the probe and its closest neighbours, divided by 2.

TEM experiments

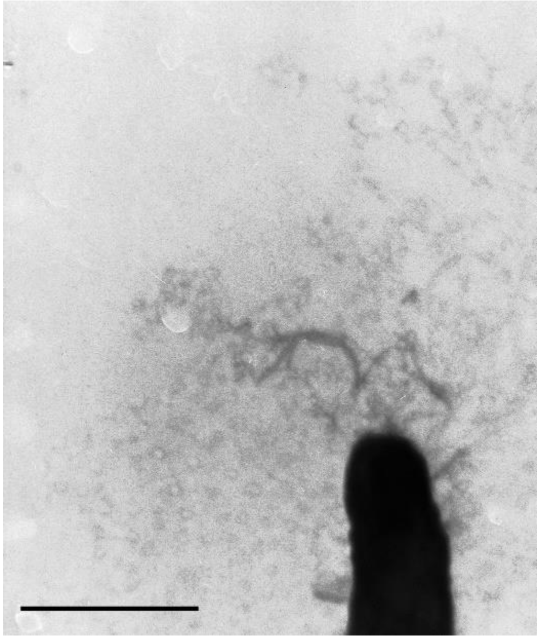

Figure S7. TEM image of the STC sample at a concentration of 2.5 × 10^{-5} M in water (stained with dipotassium polytungstate (PWK); bar = 500 nm). The platelet-like crystal (diameter around 250 nm), very tiny elongated fibers (d values of 6–12 nm) and a lot of small aggregates dimensions less than 10 nm.

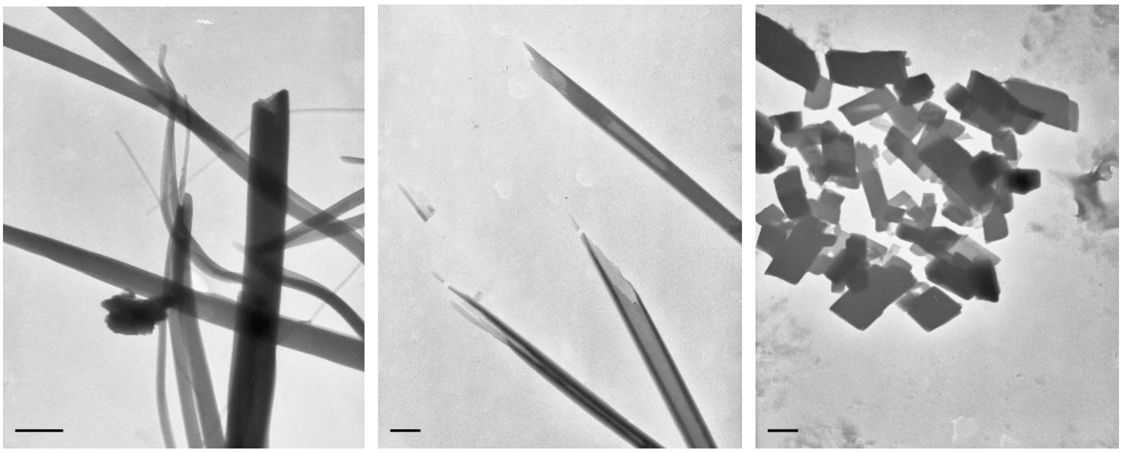

Figure S8. TEM images of the STC acetonitrile/water mixture (H_2O:CH_3CN = 2:1, *c* = 3 × 10^{-3} M, stained with dipotassium polytungstate (PWK); bar = 500 nm); nanotubules with d values of 60–500 nm (left, centre) and platelet like crystals (right).

DLS Measurements

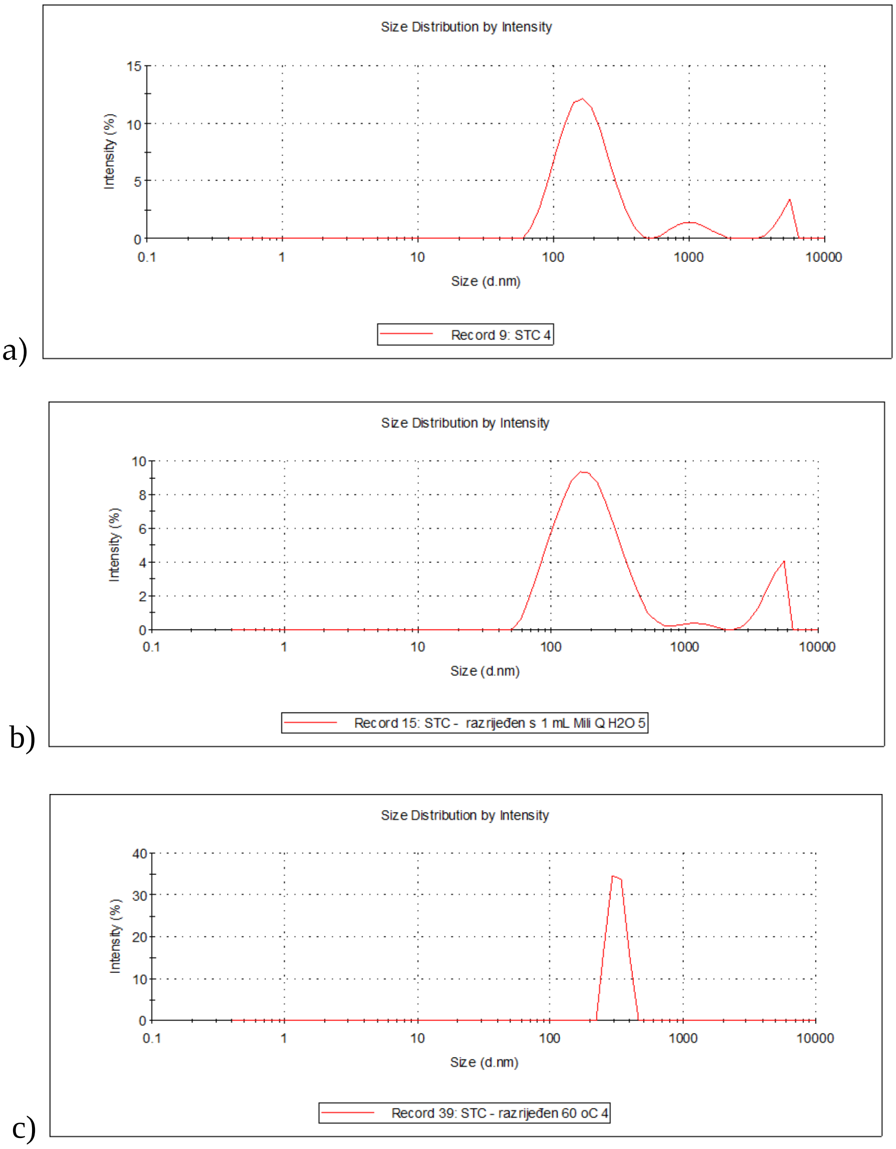

Figure S9. The STC concentration in water: (**a**) $c = 1.3 \times 10^{-5}$ M, room temp.; (**b**) $c = 5 \times 10^{-6}$ M, room temp. (**c**) $c = 5 \times 10^{-6}$ M, 60 °C.

NMR experiments

Table S1. ^{1}H NMR chemical shifts (δ/ppm) of 0.25 mM STC in various acetonitrile-water mixtures.

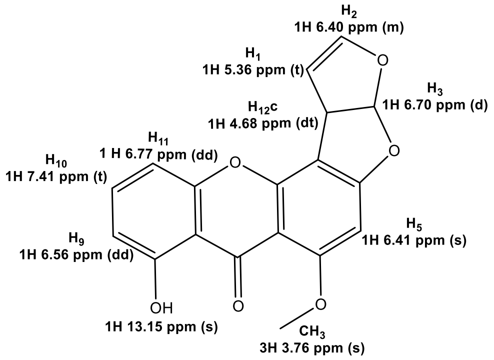

Assignment in CD_3CN.

		H-1	H-2	H-3a	H-5	H-9	H-10	H-11	H-12c	OCH_3	OH
**0.6 ml CD_3CN**	δ/ppm	5.36	6.40	6.70	6.41	6.56	7.41	6.77	4.68	3.76	13.15
**0.54 ml CD_3CN 0.06 ml D_2O**	δ/ppm	5.28	6.32	6.63	6.34	6.49	7.34	6.71	4.60	3.67	8.16
**0.45 ml CD_3CN 0.15 ml D_2O**	δ/ppm	5.14	6.19	6.50	6.21	6.36	7.22	6.58	4.46	3.53	8.01
**0.30 ml CD_3CN 0.30 ml D_2O**	δ/ppm	4.94	5.99	6.29	6.02	6.17	7.03	6.39	4.26	3.32	7.79
**0.15 ml CD_3CN 0.45 ml D_2O**	δ/ppm	4.78	5.80	6.12	5.87	6.00	6.86	6.24	4.10	3.13	7.59
**0.06 ml CD_3CN 0.54 ml D_2O**	δ/ppm	-	-	-	-	-	-	-	-	3.04	7.49
**0.03 ml CD_3CN 0.57 ml D_2O**	δ/ppm	-	-	-	-	-	-	-	-	-	7.47

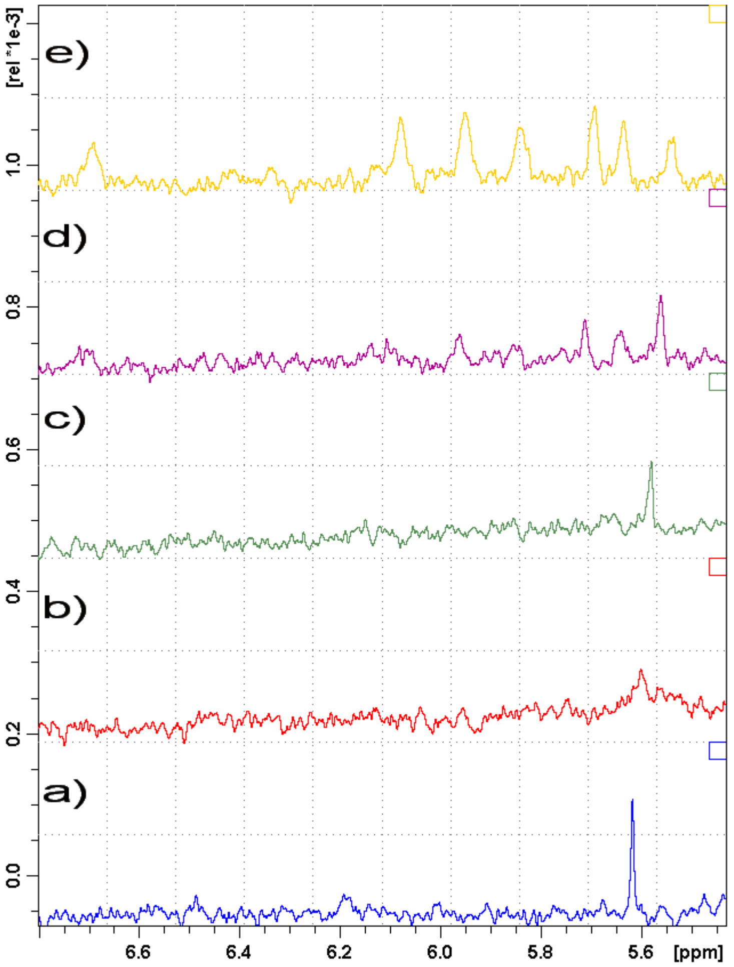

Figure S10. ^{1}H NMR spectra of 0.25 mM STC in 0.06 mL CD_3CN + 0.54 mL D_2O at: (**a**) 25 °C, (**b**) 40 °C, (**c**) 50 °C, (**d**) 60 °C and (**e**) 80 °C.

Interactions of STC with ds-DNA

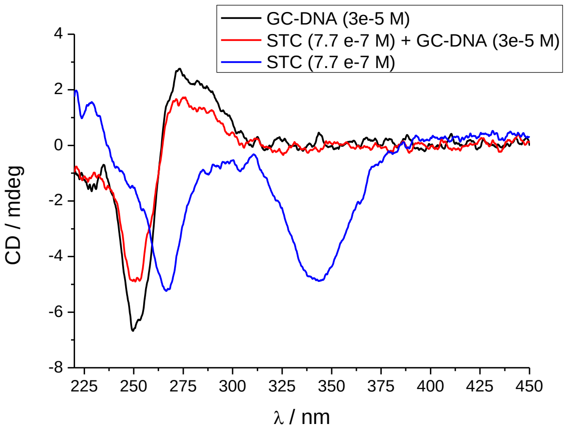

Figure S11. Comparison of CD spectra of STC water aggregate before DNA addition (blue trace), free GC-DNA (black trace) and combined STC and DNA (red trace) at large excess of DNA (r[STC]/[DNA] = 0.025).

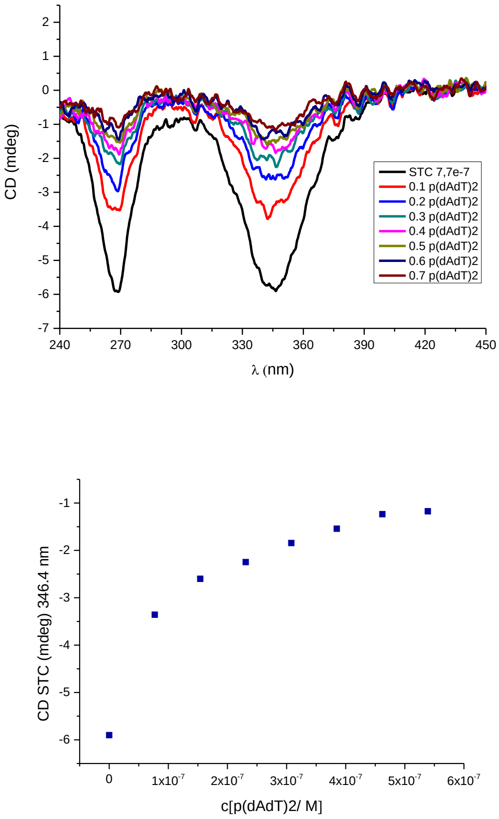

Figure S12. Left: CD titration of STC (7.7×10^{-7} M) with AT-DNA (ratios r[STC]/[DNA] = 0.1 to 0.7) at pH 7.0, sodium cacodylate buffer, I = 0.05 M, 25 °C. Right: dependence of STC CD 346 nm maximum intensity upon titration with AT-DNA.

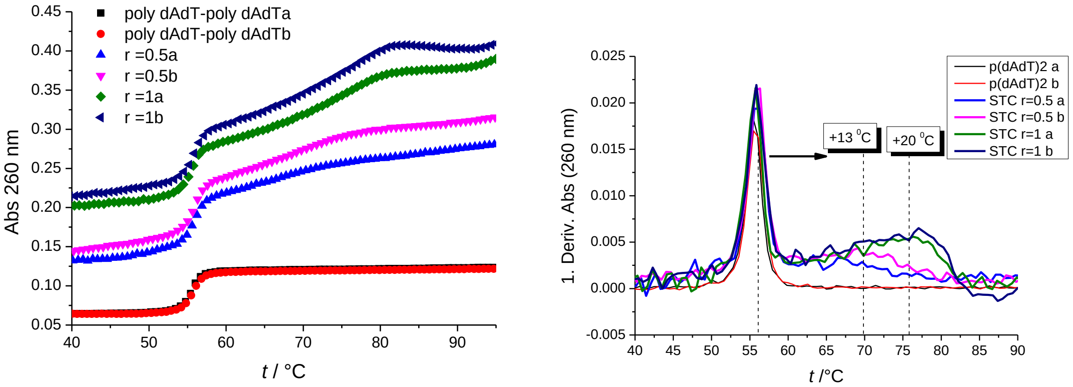

Figure S13. Left: Thermal denaturation of AT-DNA (2×10^{-5} M) in a free solution (red and black traces) and in the presence of STC (ratios r[STC]/[DNA] = 0.5 and 1) at pH 7.0, sodium cacodylate buffer, I = 0.05 M. Right: the first derivation of thermal denaturation curves on the left.

References for the Supplementary Materials

1. K. Fukuyama, K. Hamada, T. Tsukihara, Y. Katsube, T. Hamasaki and Y. Hatsuda, B Chem Soc Jpn, 1976, 49, 1153–1154

2. a) G. Pescitelli, Chirality, 24:718–724 (2012); (b) D. Padula, S. Di Pietro, M. A. M. Capozzi, C. Cardellicchio, G. Pescitelli, Chirality, 26:462–470 (2014).
